# Validation of Sport Anxiety Scale-2 (SAS-2) among Polish athletes and the relationship between anxiety and goal orientation in sport

**DOI:** 10.1038/s41598-022-16418-6

**Published:** 2022-07-19

**Authors:** Maciej Tomczak, Paweł Kleka, Aleksandra Walczak, Łukasz Bojkowski, Jacek Gracz, Małgorzata Walczak

**Affiliations:** 1 Department of Psychology, Poznan University of Physical Education, Poznań, Poland; 2 Faculty of Psychology and Cognitive Sciences, Adam Mickiewicz University in Poznan, Poznań, Poland; 3 Heliodor Święcicki Clinical Hospital of the Poznan University of Medical Sciences, Poznań, Poland; 4 Jan Amos Komeński University of Applied Sciences in Leszno, Leszno, Poland

**Keywords:** Psychology, Human behaviour

## Abstract

This study aims to assess the validity and reliability of the Polish version of the Sport Anxiety Scale-2, as well as to determine the relationship between anxiety and goal orientation among high-performance and recreational athletes. A total of 519 athletes aged M = 22.83 (SD = 4.92) participated in the study, including 266 males and 253 females. 242 athletes trained professionally and 277 recreationally. The Sport Anxiety Scale-2 (SAS-2) was used to assess anxiety levels, while the Task and Ego Orientation in Sport Questionnaire (TEOSQ) and the Perception of Success Questionnaire (POSQ) enabled to assess athletes’ goal orientation. Confirmatory factor analysis showed a good fit of the model to the data for the Polish version of the Sport Anxiety Scale-2 (CFI = 0.945, RMSEA = 0.072). The models obtained during analysis of high-performance and recreational athletes, women and men, also presented a satisfactory fit to the data (CFI 0.932–0.946). The configural, metric, scalar and strict measurement invariances were demonstrated for high-performance and recreational athletes as well as among women and men. High internal consistency coefficients (alpha 0.81–0.91) and a high test–retest reliability indexes were reported (ICC 0.74–0.87). Women presented higher level of competitive anxiety than men. A positive relationship between competitive anxiety and athletes’ ego orientation was also presented. This relationship concerned particularly women practicing sport recreationally.

## Introduction

Among the determinants of effective functioning in sport, emotional factors occupy a vital place. The importance of anxiety is often emphasized here. Anxiety generally manifests itself as nervousness and fears connected with some anticipated (not necessarily accurate) threat, which may be accompanied by increased emotional and muscular tension, insomnia, decreased efficiency of cognitive processes e.g., problems with attention or logical thinking^1,2^. In sports competition, two dimensions of anxiety (the so-called competitive anxiety) are often considered – the somatic and the cognitive component^3,4^. The somatic aspect concerns the athletes’ bodily reactions (e.g., muscle tension, stomach problems, hand tremors) that they may experience before and/or during participation in sports competition. The cognitive component, on the other hand, is manifested, among other things, by problems with concentration and effective thinking, as well as anxiety about how to cope successfully with athletic competition^3–6^.

With the growing demand for accurate and reliable diagnosis of sport anxiety, several scales have been developed, referring to different definitions and theories of anxiety. Frequently used scales include: Sports Competition Anxiety Test (SCAT^3^), Competitive State Anxiety Inventory (CSAI-2 and CSAI-2R^4,7^) and Sport Anxiety Scale (SAS and SAS-2^8,9^)^6^. The Sport Anxiety Scale-2 (SAS-2), due to its high validity and reliability indices as well as the potential ease and speed of taking the survey – is one of the most widely used tools. Developed by Smith et al.^9^, the SAS-2 was constructed using a theoretical strategy of questionnaire construction and its theoretical basis is the cognitive-affective model. The areas of the scale relate to the aforementioned cognitive and somatic components of anxiety. In light of the data obtained in studies which used the previously constructed Sport Anxiety Scale, Smith et al.^9^ aimed to create a relatively short, yet accurate and reliable tool for adult and young athletes. Three dimensions were assumed to be present: somatic anxiety, worry and concentration disruption. After a pre-test conducted on the young athletes in order to detect the understanding of the items, ten items were extracted for each of the three components. Then, five items from each dimension were culled by exploratory factor analysis. Finally, the 15-item scale achieved high reliability indices and a good fit of the theoretical model to the data in each of the extracted age groups^9^. Satisfactory validity and reliability indices of the scale were also confirmed in studies conducted in different countries, e.g., Spain^10^, Brazil^6,11^, Malaysia^12^, U.S.^13^ and Korea^14^.

It is worth considering that the SAS-2 scale is used to measure competitive anxiety as a trait as opposed to an anxiety state^9^. Thus, anxiety measured by the SAS-2 scale expresses relatively constant and stable tendency to experience anxiety in situations before and during athletic competition. In contrast, anxiety as a state would refer to a psychological and physiological response, e.g., during a specific competition, and would be temporary in nature. When measuring anxiety as a trait, subjects are asked to recall the anxiety symptoms they usually experience, whereas when measuring anxiety as a state, subjects’ responses refer to anxiety symptoms at a specific point in time^15,16^. It is also worth noting, that in light of the assumptions of the scale design^9^, the SAS-2 examines the intensity of an anxiety trait, where it is assumed that high levels of the trait may have negative consequences. However, anxiety does not always have to cause a negative impact on performance in sport. Referring to this aspect, the concept of “directionality of anxiety” was introduced^17–19^. It was assumed that the person’s ability to control the stressor impacts further competence to interpret anxiety symptoms. They can be interpreted as facilitative or debilitative for performance. With reference to these assumptions, as well as further development of the “directionality of anxiety” concept, positive interpretation of anxiety symptoms may increase the chance of high sport performance. The factors determining the interpretation of anxiety symptoms include e.g., self-confidence, personality traits (mainly neuroticism and extraversion), coping strategies, cognitive abilities, sport level as well as situational factors^20,21^. Moreover, it is worth noting that the SAS-2 measuring anxiety as a trait does not take into account the “directionality of anxiety”. However, the scale has a high diagnostic value for psychological assessment in sport area. It has been confirmed in many validity and reliability studies. For example, the SAS-2 scale factorial validity verified by Confirmatory Factor Analysis was demonstrated^9,10,13,14^. Convergent validity was confirmed by the relationships of the SAS-2 scale scores with other scales dedicated to measure anxiety in sport area as well as general anxiety^6,12,13^. Moreover, the discriminant validity of the SAS-2 scale was also indicated, which is important from the perspective of clinical diagnosis in sport. In the latter aspect, Silva-Rocha et al.^6^ analyzing specificity and sensitivity indices, identified cut-off points of the SAS-2 for differentiating athletes in terms of general anxiety as a trait, social anxiety and even depressive symptoms. Based on this analysis, the authors suggested that the SAS-2 scale can also be used for initial identification of athletes with psychopathological indicators^6^. The value of SAS-2 scale was also highlighted by other scientific research on the relationship of scale scores with other variables relevant to activity in sport, which will be briefly presented. Hence, the SAS-2 scale is still very popular worldwide as a scale measuring anxiety for athletes.

The associations of anxiety with psychological factors necessary for functioning in sports have been demonstrated in the study validating the relevance of the SAS-2 scale as well as in separate studies on different topics. For example, Smith et al.^9^ in validation studies of the SAS-2 scale showed negative relationships between all components of anxiety and self-esteem. Moreover, positive, although small, relationships were found between all components of anxiety and ego goal orientation. On the other hand negative relationships between somatic anxiety and concentration disruption and task goal orientation were observed. Similar results were obtained when examining the associations of anxiety with motivational climate, where the mastery factor correlated negatively, whereas ego climate correlated positively with anxiety. Small negative association of anxiety with social desirability was also found^9^. Negative association of overall anxiety score with self-esteem was also obtained by Huđin et al.^22^, who also showed a positive association of anxiety with aggressiveness and the small positive, associations of anxiety with competitive aggressiveness and anger. Furthermore, the study by Kalinin et al.^23^ also reported association of anxiety with mental toughness in sport (in case of handball players). The worry and the concentration disruption subscales were negatively associated with confidence, constancy and control. On the other hand, somatic anxiety was negatively associated with control and, to a smaller extent, with constancy^23^. Additionally, high levels of anxiety were shown to be associated with less effective coping strategies in sport (among hockey players), in particular, factors such as freedom from worry, coping with adversity and coachability, concentration and peaking under pressure^24^.

Associations of anxiety as measured by the SAS-2 scale with non-psychological factors, but relevant from the perspective of functioning in sporting activities, were also assessed. For example, Grossbard et al.^25^ showed significant, although small associations of anxiety with age and gender of children participating in sport. Older children obtained higher levels of the worry factor than younger children. In addition, girls presented higher levels of worry and lower levels of concentration disruption than boys. Moreover, Coreira and Rosado^26^ showed that females had higher levels of general anxiety, somatic anxiety and concentration disruption than males and individual athletes had higher levels of anxiety than team athletes on the same subscales. Furthermore, in a predictive model for anxiety, Ramis et al.^5^ showed that women had slightly higher levels of worry than men. The score on this subscale was positively associated with the age of the athletes. It was also shown that individual athletes had higher levels of somatic anxiety than team athletes. However, the noted effects were not large.

The aim of our work was to analyze the validity and reliability of the Polish version of the Sport Anxiety Scale-2. The availability of the SAS-2 scale in Poland would significantly expand the diagnostic possibilities of anxiety among athletes. An additional aim of the study was the assessment of association between anxiety and goal orientation in athletes, which was also presented by the authors of the SAS-2 scale^9^. Goal orientation was derived from the Achievement Goal Theory (AGT)^27,28^, which assumes that people, while performing performance tasks, may be oriented towards task completion (task orientation) or comparing themselves with others (ego orientation). According to AGT task-oriented athletes consider their own progress in the task as success, whereas ego-oriented athletes consider the fact that they perform the task better than other athletes as success^27–30^. In a validation study of the SAS-2 scale, Smith et al.^9^ obtained negative association of anxiety with task orientation and positive association with ego orientation. Fairly similar results were obtained by Grossbard et al.^25^ in the study of young athletes. comparable results were also obtained by Duica et al.^31^. The addressed problem concerning the relationship between goal orientation and anxiety may be important from the perspective of sports practice because it is possible that the coach’s actions (strengthening or weakening a specific goal [ego/task]) orientation of athletes may be, to some extent, connected with experiencing specific emotions, e.g., anxiety before and during competition. It’s possible that activating an ego-oriented motivational climate during a training process simultaneously promotes an increase in competitive anxiety, whereas a task-oriented climate promotes a decrease in anxiety^9^. For example, task-oriented individuals may experience less anxiety in competitive situations because their sense of competence, based on an internal criterion for success, is not threatened by their performance compared to other athletes^32^. However, taking into account the character of correlational research, another direction of the relationship cannot be ruled out^9^, e.g., that individuals with higher levels of anxiety may be characterized by higher emotional arousal of behavior, which reduces concentration on the task (task orientation), while by reducing control (increased arousal) it increases confrontational behavior (ego orientation).

In addition, proceedings of our study took into account gender and level of participation in sport (high-performance, recreational). It was also worth determining whether there was a difference between competitive and recreational athletes because these groups differ in a number of factors e.g., motives for taking up sport activities, expectations of sport performance, personality traits etc.^33–35^. In light of previous research findings, comparing these groups in terms of anxiety may be relevant. As indicated in the review on the importance of personality for sport functioning^35^, professional elite athletes are characterized by greater emotional stability (strongly associated with lower anxiety) compared to recreational athletes. These results are also supported by a recent study on volleyball players, where professional athletes were characterized by lower levels of anxiety as a trait than amateurs^36^. Therefore, it can be assumed that low levels of anxiety are conducive to effective performance in competitive and stressful professional sport. However, the prediction of differences in anxiety across our researched groups is not entirely clear, as the SAS-2 scale measures generalized levels of anxiety before and during competition^9^. In the research conducted by Marcel and Paquet^37^ where an older version of the SAS scale was used the results showed that athletes with high athletic level were characterized by higher anxiety than less advanced athletes. Differences here may be due to different approaches to sports competition and expectations concerning sports performance in these groups, e.g., professional athletes may have a much higher level of expectations concerning sports performance, which may intensify the experience of anxiety. However, in our SAS-2 scale validation, even more important than the analysis of differences in the levels of anxiety may be the assessment of invariance of anxiety measurement in the groups of professional and recreational athletes^38^. To our knowledge, neither such analyses exist for anxiety measurement by the SAS-2 scale, nor has a study been conducted on the relationship between anxiety and goal orientation among competitive and recreational athletes separately.

The growing popularity of sport psychology in Poland in recent years has been visible in the educational offer in this field. Several Polish universities provide specializations or postgraduate studies in sport psychology. Coaches and athletes cooperate with psychologists more often. In the latter aspect, it is worth noting that the basis for effective psychological intervention in sport is an accurate diagnosis. Despite the validation of various internationally known sports psychology scales into the Polish cultural context, there is still a need to adapt new ones. Therefore, validation of the SAS-2 scale will significantly broaden the possibilities of anxiety measurement among Polish athletes. The value of Polish validation is also enhanced by the possibility to use the scale not only on professional but also on recreational athletes. There are not many adaptations in the Polish cultural context which take into account a separate division into these groups of athletes. Moreover, a new tool in sport psychology such as the SAS-2 scale increases possibilities of searching for relations between anxiety and other variables, important from the theoretical and empirical point of view, which have not been verified before.

## Methods

### Participants

The study included 519 athletes aged M = 22.83 (SD = 4.92), 266 men and 253 women, 242 athletes trained professionally and 277 recreationally. The disciplines that the respondents practiced most often were: football, running, combat sports, swimming, volleyball, basketball, dancing, handball. All of the surveyed high-performance athletes were affiliated with professional sports clubs and reported that they trained in sports primarily for maximum results, moreover they systematically participated in national level competitions. Recreational athletes were not affiliated with professional sports clubs however participated in recreational athletic activities with competition and competed sometimes in sporting events (e.g., individual regional running competitions or academic competitions). They specified that they exercised mainly for health, physical fitness, and appearance. Due to the anonymity of the study, this information was obtained through a self-administered metric completed by respondents.

### Measurement tools

The Sport Anxiety Scale-2^9^ consists of 15 items which form 3 subscales (somatic anxiety, worry and concentration disruption), 5 items each, and was used to investigate anxiety. The questionnaire instruction highlights that many athletes feel tense or experience nervousness before or during competitions and games. The respondents are asked to mark the number (1-not at all to 4-very much) that most closely corresponds to how they usually feel during or before sport competition. At the beginning of the adaptation procedure, an English language expert translated the SAS-2 scale into Polish, and then another expert translated the scale back into English. Finally, the final version of the scale was agreed upon by a group of experts consisting of three sport psychologists (including two coaches) and an English language expert. At the beginning of the study, the subjects were asked about doubts in understanding the statements or instructions. Nobody made any comments in this respect. The research was completely anonymous and the survey was conducted via the internet (online questionnaire). After reviewing the study description, the Bioethics Committee of Poznan University of Medical Science (Poland) issued a statement that the study had no features of a medical experiment and according to the Polish law and Good Clinical Practice it was not subject to the opinion of the Bioethics Committee. All methods were carried out in accordance with relevant guidelines and regulations. The research was performed in accordance with the Declaration of Helsinki. The study instructions included information that taking part in the research and submitting the results would be treated as the respondent’s informed consent to take part in the research.

The Task and Ego Orientation in Sport Questionnaire—TEOSQ^29,39^ and the Perception of Success Questionnaire—POSQ^40^ in the Polish adaptation^41,42^ were used to measure goal orientation. The TEOSQ consists of 13 statements; 7 items concern task orientation and six items concern ego orientation. The respondent determines to what extent a given statement applies to him/her on a scale of 1–5. The POSQ, on the other hand, consists of 12 statements, 6 for each goal orientation (ego, task), where the respondent’s task is also to assess to what extent he/she agrees with a given statement on a scale of 1–5 (A–E).

### Statistical analysis

First, to assess construct validity confirmatory factor analysis was conducted. According to theoretical assumptions, a hierarchical model was estimated with a general factor of anxiety (overriding factor) and three subscales: somatic anxiety, worry and concentration disruption. Next, the 3-factor model without the overriding factor of anxiety was estimated, taking into account the three subscales. Additionally, in order to check the validity of extracting the subscales of the tool, the 1-factor model (without extracting the subscales of the tool) was estimated and compared with two models, including the subscales. CFI and TLI above 0.90, RMSEA and SRMR below 0.08 were assumed to indicate a satisfactory fit of the postulated models to the data^43^. Due to deviations from multivariate normality of the distribution, the correction proposed by Satorra-Bentler^44^ was applied.

Additionally, scale invariance by gender and type of sport participation (high performance, recreational) was examined. First, a model for configural invariance was estimated, then for metric invariance, where factor loadings within groups were fixed. Then a model for scalar invariance was estimated, where regression intercepts were additionally fixed, and finally a model for strict invariance, where residuals within comparison groups were fixed. It was assumed that a decrease in CFI below 0.01 and an increase in RMSEA above 0.015 indicate significant differences within groups. For SRMR, an increase of 0.01 for scalar and strict invariance, and 0.03 for metric invariance, indicated significant group differences^38^.

Convergent validity indices of the SAS-2 scale were also analyzed. Item reliability was assessed by analyzing the values of factor loadings and their values squared (the proportion of variance in an item due to a given construct). In general, criteria ranging from 0.40 (most liberal) to 0.70 (close to 50% of the variance in the item explained by the factor) are adopted for minimum standardized loadings values^45^. Considering these criteria, as well as the analysis of factor loading values presented in SAS-2 validation articles conducted in other countries, we considered that a minimum standardized loading value of 0.5 would be sufficient. Average variance extracted (AVE) and composite reliability (CR) values were then calculated for each subscale^46^. It is conventionally accepted that an AVE value above 0.5 and a CR value above 0.6 (or 0.7 under a more restrictive assumption) indicates sufficient convergent validity. However, it is also often assumed that a construct meets the requirement of convergent validity when AVE is below 0.5 but composite reliability (CR) requirement is fulfilled^46^. Discriminant validity, on the other hand, was assessed by comparing the AVE of a given latent variable with the maximum shared variance (MSV) (the greatest value of the square of the correlation of a given latent variable with another construct-latent variable). The discriminant validity of the construct is confirmed when the AVE value is higher than the MSV^46^.

The reliability of the scale was determined by calculating Cronbach’s alpha and McDonald’s omega coefficients, as well as the ICC between two measurements of the same athlete at a two-week interval (N = 189, all athletes completed the scale twice). The discriminatory power of a particular test item was calculated by its correlation with the total subscale score.

A two-factor analysis of variance was used to compare individuals by gender and level of sport participation in terms of mean anxiety. The r-Pearson correlation coefficient was used to assess the relationship between anxiety and goal orientation (N = 312).

## Results

### Construct validity: confirmatory factor analysis

**Figure 1:**
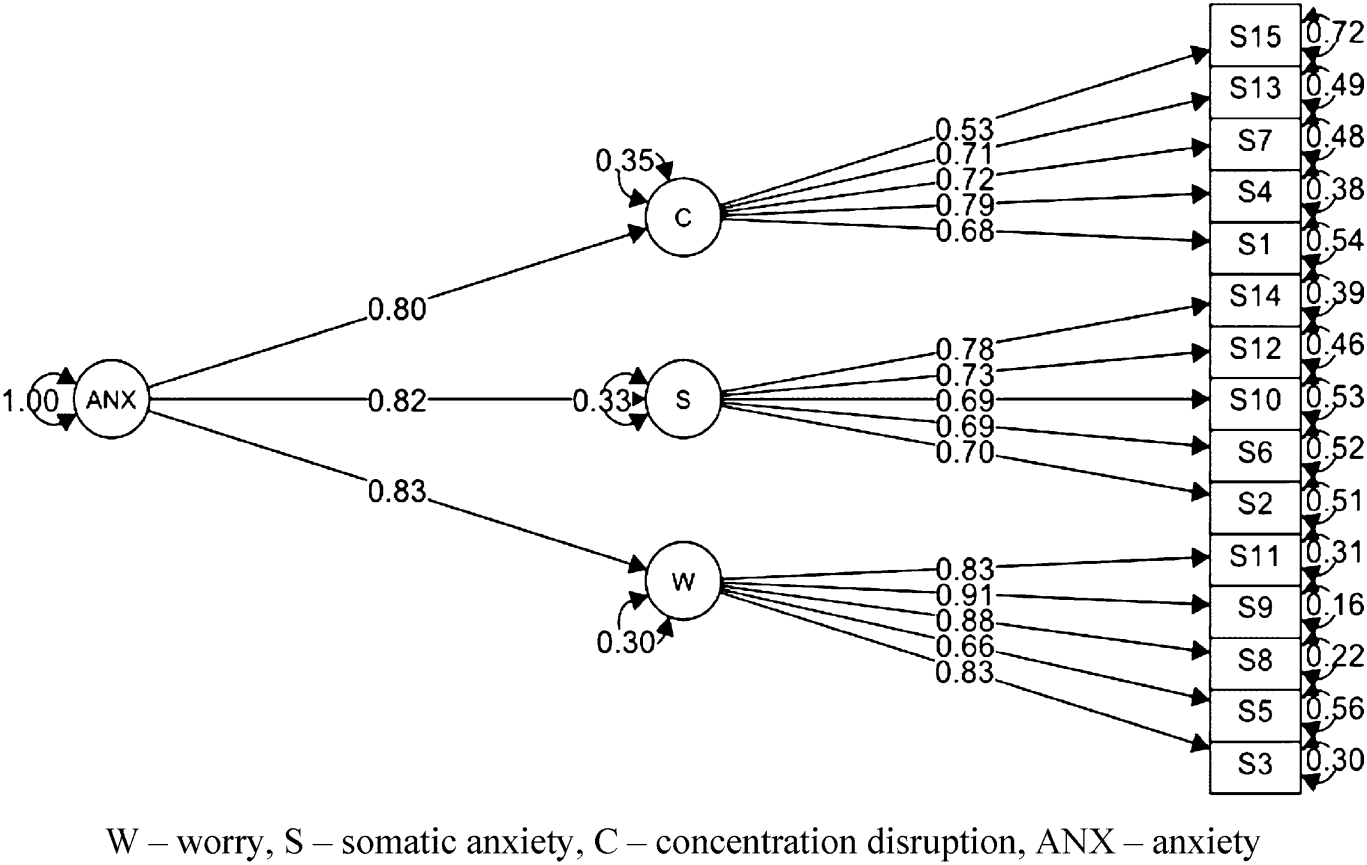
Hierarchical CFA model for the Polish version of the Sport Anxiety Scale-2.

**Figure 2:**
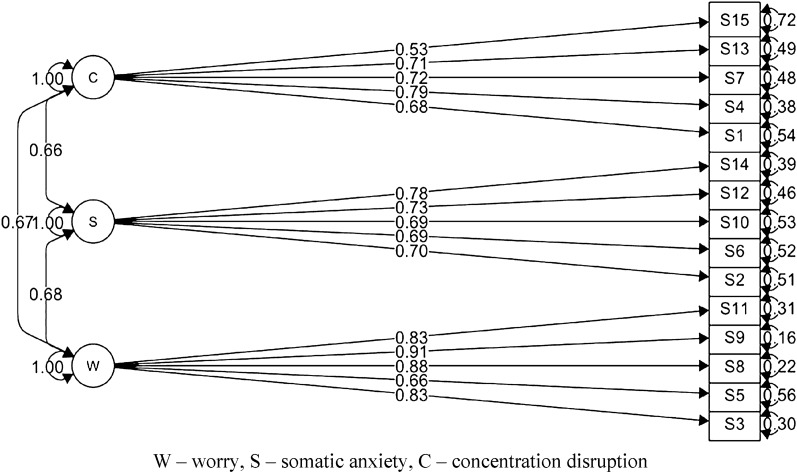
A 3-factor CFA model for the Polish version of the Sport Anxiety Scale-2.

As indicated by the data in Table 1, both the hierarchical model (Fig. 1) and the 3-factor model (Fig. 2) fit the empirical data well. They had much better fit than the 1-factor model, which did not obtain an adequate fit to the data. The 3-factor model also fit the data well within gender and in the group of high performance and recreational athletes (Table 1).

**Table 1:** CFA models for Polish version of the Sport Anxiety Scale-2.

Models (N = 519)	Chi-square (df)	*p*	CFI	TLI	RMSEA 90% CI	SRMR
1-factor	838.916 (90)	< 0.001	0.775	0.738	0.143 [0.135, 0.152]	0.092
Hierarchical	280.095 (87)	< 0.001	0.945	0.933	0.072 [0.063, 0.082]	0.046
3-factor	280.095 (87)	< 0.001	0.945	0.933	0.072 [0.063, 0.082]	0.046
Male	185.847 (87)	< 0.001	0.932	0.918	0.074 [0.059, 0.088]	0.058
Female	186.254 (87)	< 0.001	0.946	0.935	0.073 [0.058, 0.087]	0.049
High-performance	169.526 (87)	< 0.001	0.935	0.922	0.068 [0.053, 0.083]	0.057
Recreational	206.101 (87)	< 0.001	0.945	0.934	0.079 [0.065, 0.093]	0.051

CFI, Comparative Fit Index; TLI, Tucker–Lewis Fit Index; RMSEA, Root Mean Square Error of Approximation; SRMR, Standardized Root Mean Square Residual - robust values based on Satorra-Bentler correction.

Factor loadings of the SAS-2 scale model achieved statistical significance (*p* < 0.001) and range from 0.53 to 0.91 (Fig. 2). Thus, squared multiple correlations for the worry scale were respectively: 3–0.69, 5–0.44, 8–0.77, 9–0.83, 11–0.69; for the somatic anxiety scale: 2–0.49, 6–0.48, 10–0.48, 12–0.53, 14–0.61; for the concentration disruption scale: 1–0.46, 4–0.62, 7–0.52, 13–0.50, 15–0.28. The average variance extracted (AVE) and composite reliability (CR) values were: AVE = 0.68, CR = 0.91 for the worry scale; AVE = 0.52, CR = 0.84 for the somatic anxiety scale; and AVE = 0.48, CR = 0.82 for the concentration disruption scale, respectively. For each subscale, the AVE value was higher than the maximum shared variance (MSV) value, which were for the worry scale: MSV = 0.46, for the somatic anxiety scale: MSV = 0.46, for the concentration disruption scale: MSV = 0.45, respectively (squares of correlations between latent variables for the 3-factor model from Fig. 2). This allowed us to assume discriminant validity of the tested constructs.

### Invariance across gender and level of participation

The postulated models fit well to the data. The decrease in CFI greater than 0.01 and the increase in RMSEA greater than 0.015 were not observed in any case. Hence, measurement invariance of the SAS-2 scale can be assumed both across gender and level of sport participation. Analysis of the SAS-2 invariance by gender and level of participation is presented in Table 2.

**Table 2:** Analysis of the Sport Anxiety Scale-2 invariance by gender and level of participation.

	CFI	RMSEA	SRMR	ΔCFI	ΔRMSEA	ΔSRMR
**Gender (males vs females)**						
Configural	0.940	0.073	0.050	–	–	
Metric	0.941	0.070	0.053	0.001	0.003	0.003
Scalar	0.938	0.069	0.056	0.003	0.001	0.003
Strict	0.936	0.068	0.059	0.002	0.001	0.003
**Level of participation (high-performance vs recreational)**						
Configural	0.942	0.074	0.051	–	–	
Metric	0.941	0.072	0.057	0.001	0.002	0.006
Scalar	0.937	0.072	0.059	0.004	0.000	0.002
Strict	0.941	0.068	0.059	0.004	0.004	0.000

CFI, Comparative Fit Index; TLI, Tucker-Lewis Fit Index; RMSEA, Root Mean Square Error of Approximation; SRMR, Standardized Root Mean Square Residual- robust values based on Satorra-Bentler correction.

### Reliability and discriminatory powers of the Polish version of the Sport Anxiety Scale-2

The Cronbach alpha coefficient for the worry subscale was 0.91 (ω = 0.91), and the discriminant power coefficients for the individual items were respectively: 3–0.79, 5–0.64, 8–0.82, 9–0.85, 11–0.78. The Cronbach’s coefficient for the somatic anxiety subscale was 0.84 (ω = 0.83), and the discriminant power coefficients were respectively: 2–0.61, 6–0.64, 10–0.61, 12–0.67, 14–0.70. The coefficient for the concentration disruption subscale was 0.81 (ω = 0.81), and the item-scale correlations were respectively: 1–0.58, 4–0.68, 7–0.66, 13–0.64, 15–0.46. The coefficient alpha for the whole scale was 0.92 (ω = 0.92).

Interclass correlations (ICC) between two measurements with two weeks interval were: total anxiety: ICC = 0.87; somatic anxiety: ICC = 0.87; worry: ICC = 0.80; concentration disruption: ICC = 0.74.

### Relationships between gender and level of participation in sport and anxiety

There was a statistically significant effect of gender for the total anxiety score: F(1,515) = 36.29; *p* < 0.001; ${\eta }_{p}^{2}$ = 0.066, for worry factor: (F(1,515) = 40.72; *p* < 0.001; ${\eta }_{p}^{2}$ = 0.073; for somatic anxiety: F(1,515) = 29.33; *p* < 0.001; ${\eta }_{p}^{2}$ = 0.054; and for concentration disruption: F(1,515) = 7.62; *p* < 0.01; ${\eta }_{p}^{2}$ = 0.015. Men had lower mean anxiety levels then women. However, there was no statistically significant effect of level of participation (high performance, recreational) on overall anxiety: F(1,515) = 0.26; *p* > 0.05, for worry: F(1,515) = 1.23; *p* > 0.05, for somatic anxiety: F(1,515) = 1.52; *p* > 0.05, and for concentration disruption: F(1,515) = 3.10; *p* > 0.05. There were also no statistically significant interaction effects of gender and participation level for the total score: F(1,515) = 0.04; *p* > 0.05, for worry factor: F(1,515) = 0.28, *p* > 0.05, for somatic anxiety: F(1,515) = 0.02; *p* > 0.05, and for concentration disruption: F(1,515) = 0.01; *p* > 0.05. Descriptive statistics for female, male, high-performance and recreational athletes are presented in Table 3.

**Table 3:** Descriptive statistics for the Polish version of the SAS-2 scale.

Sex LP		N	W M (SD)	S M (SD)	C M (SD)	A M (SD)
–	–	519	10.53 (3.71)	8.89 (3.04)	7.28 (2.35)	26.71 (7.76)
F	–	253	11.56 (3.83)	9.63 (3.21)	7.59 (2.46)	28.78 (8.16)
M	–	266	9.56 (3.31)	8.19 (2.69)	6.99 (2.21)	24.74 (6.81)
R	–	277	10.45 (3.95)	9.10 (3.28)	7.47 (2.61)	27.02 (8.74)
HP	–	242	10.63 (3.40)	8.66 (2.73)	7.06 (2.00)	26.35 (6.46)
F	R	146	11.48 (4.06)	9.75 (3.45)	7.75 (2.63)	28.98 (9.06)
F	HP	107	11.66 (3.51)	9.47 (2.87)	7.37 (2.20)	28.50 (6.79)
M	R	131	9.30 (3.50)	8.37 (2.93)	7.17 (2.56)	24.84 (7.84)
M	HP	135	9.81 (3.09)	8.01 (2.43)	6.81 (1.80)	24.64 (5.66)

W, worry; S, somatic anxiety; C, concentration disruption; A, anxiety (total score); F, female; M, male; R, recreational; HP, high-performance; LP, level of participation.

### Correlations between competitive anxiety and goal orientation among high-performance and recreational athletes

Statistically significant, negative small associations were found between anxiety (general and subscales: somatic anxiety and concentration disruption) and ego orientation (TEOSQ subscale) in the whole group of the examined athletes. Correlations between anxiety and athletes’ goal orientation are presented in Table 4. Moreover, significant negative associations were found between general anxiety and the subscales of somatic anxiety and ego orientation in the group of women and in the group of recreational athletes. In the case of recreationally training women, the relationships were stronger and concerned both the TEOSQ and the POSQ. The positive correlation between concentration disruption and ego orientation assessed with TEOSQ was also noted in the group of high-performance male athletes. Correlations between anxiety and goal orientation in males and females, recreational and high-performance athletes are presented in Table 5.

**Table 4:** Correlations between anxiety and athletes’ goal orientation.

Variable	N = 312			
	W	S	C	A
E TQ	0.07	0.14*	0.12*	0.12*
T TQ	0.03	0.10	− 0.07	0.03
E PQ	0.01	0.06	0.06	0.05
T PQ	0.01	0.09	− 0.03	0.03

W, worry; S, somatic anxiety; C, concentration disruption; A, anxiety (total score); E TQ, T TQ, ego and task assessed with the TEOSQ; E PQ, T PQ, ego and task assessed with the POSQ.

*p < 0.05.

**Table 5:** Correlations between anxiety and goal orientation in males and females, recreational and high-performance athletes.

Variable	Males (N = 143)				Females (N = 169)			
	W	S	C	A	W	S	C	A
E TQ	0.06	0.11	0.15	0.12	0.14	0.23**	0.13	0.20**
T TQ	− 0.13	0.04	− 0.16	− 0.10	0.09	0.06	− 0.04	0.05
E PQ	0.02	0.04	0.06	0.05	0.10	0.18*	0.10	0.15*
T PQ	− 0.12	0.01	− 0.12	− 0.09	0.03	0.09	0.02	0.06

	High performance (N = 157)				Recreational (N = 155)			
	W	S	C	A	W	S	C	A
E TQ	0.00	0.13	0.03	0.06	0.14	0.18*	0.24**	0.20*
T TQ	0.03	0.09	− 0.01	0.05	0.03	0.08	− 0.15	0.00
E PQ	− 0.10	0.04	− 0.04	− 0.05	0.10	0.16*	0.21**	0.17*
T PQ	− 0.02	0.06	− 0.07	− 0.01	0.04	0.10	− 0.01	0.05

	Female—high performance (N = 75)				Female—recreational (N = 94)			
	W	S	C	A	W	S	C	A
E TQ	− 0.00	0.17	− 0.10	0.03	0.28**	0.32**	0.35***	0.35***
T TQ	0.02	0.02	0.09	0.05	0.13	0.09	− 0.13	0.06
E PQ	0.01	0.17	− 0.08	0.05	0.18	0.24*	0.28**	0.25*
T PQ	0.01	0.02	− 0.11	− 0.03	0.05	0.13	0.13	0.11

	Male—high performance (N = 82)				Male—recreational (N = 61)			
	W	S	C	A	W	S	C	A
E TQ	0.04	0.16	0.22*	0.15	0.06	0.07	0.11	0.09
T TQ	− 0.05	0.05	− 0.15	− 0.06	− 0.22	0.01	− 0.20	− 0.15
E PQ	− 0.12	0.03	0.11	− 0.02	0.08	0.11	0.13	0.12
T PQ	− 0.10	0.02	− 0.08	− 0.07	− 0.14	− 0.01	− 0.17	− 0.12

E TQ, T TQ; ego and task assessed with the TEOSQ; E PQ, T PQ, ego and task assessed with the POSQ; W, worry; S, somatic anxiety; C, concentration disruption; A, anxiety (total score).

*p < 0.05, ** < 0.01, *** < 0.001.

## Discussion

First of all, through confirmatory factor analysis, the theoretical validity of the Polish version of the SAS-2 was examined. The model of the Polish version of the scale fit the empirical data well both for the entire group of respondents and separately in the groups of women, men, high-performance and recreational athletes. The model fit was not perfect (e.g., CFI > 0.95, TLI > 0.95), but it was sufficient to accept the SAS-2 factor validity (CFI > 0.90, RMSEA < 0.08). The hierarchical model and the 3-factor model had a significant advantage of fitting to empirical data over the single-factor model, which confirms the validity of separating the theoretically justified three subscales of the SAS-2 scale. Comparing the obtained results with the results of other authors, it can be concluded that both hierarchical and the 3-factor model of the Polish version of the scale estimated on the whole group of respondents had worse fit to the data than the models presented by the authors of the scale^9^, the models of the Spanish version of the scale^10^ and the 3-factor model of the American version of the scale^13^. In turn, the models of the Polish version of the scale obtained a better fit to the data than the model (three factor model without correlating errors for items) of the Brazilian version of the scale^6^. Based on the CFI index, the models of the Polish version of the SAS-2 also obtained a better fit to the data and based on the RMSEA, a slightly worse fit than the 3-factor Malaysian model of the scale conducted on young athletes^12^ and the Korean version of the SAS-2 (both hierarchical and the 3-factor model of the scale)^14^. High correlations were also found between the factors representing each of the anxiety subscales, which is consistent with previous research findings.

It can be assumed that the values of factor loadings obtained in the Polish validation of SAS-2 were satisfactory. The AVE values for the worry and somatic anxiety scales were satisfactory, whereas the AVE value for the concentration disruption scale was slightly below the accepted conventional threshold value (0.5). However, convergent validity can be accepted in light of the high composite reliability values obtained (for each subscale above 0.8). Moreover, AVE for each subscale was higher than MSV, which allowed us to assume discriminant validity of the tested constructs. The lower AVE for the concentration disruption subscale of the SAS-2 scale was not surprising. Even the authors of the original version of the scale^9^ obtained for this subscale lower factor loadings on average than the values obtained by us. From the data presented by Smith et al.^9^ it is easy to calculate that those authors also obtained a lower AVE value than the one obtained in our study. However, due to the theoretical importance of the selected items, we assumed that it was not worth removing any of them. Lower AVE values are not that uncommon for the SAS-2 scale. This is partly due to conservativeness of the AVE coefficient^46^ (it’s necessary that the average factor loadings value is above 0.7). For example, slightly higher AVE values for the concentration disruption subscale than the values in our study were obtained by Correira and Rosado^47^, Putra et al.^48^ and Cho et al.^14^. The two latter researchers also obtained values under 0.5 for the somatic anxiety subscale.

High Cronbach’s alpha reliability coefficients obtained in our analysis were also observed for the individual subscales and the total score, which were close to the results obtained by the authors of the original scale^9^, higher than the coefficients obtained for the Spanish version^10^ and medium higher than the coefficients obtained for the Brazilian version^6^. The high reliability of the Polish version of the SAS-2 scale was also confirmed by the high values of McDonald’s omega coefficients and fair values of inter-class correlations for the study of the same athletes at a two-week interval. The measurement was also shown to be invariant considering gender (women, men) and the level of participation in sport (high-performance, recreational). Four basic types of measurement invariance were demonstrated in both cases, i.e. configural, metric, scalar and strict. Therefore, it can be assumed that women will use the scale similarly as men, and high-performance athletes will use the scale in a similar way to recreational athletes. Measurement invariance by gender was demonstrated previously [e.g.,^5,13,25^], whereas measurement invariance in high-performance and recreational athletes brings new information.

In Polish validation of the SAS-2 scale a comparison was also made of the average level of anxiety by gender and level of participation in sport. It was shown that women are characterized by higher levels of general anxiety and its individual components compared to men. The difference obtained can be explained by the greater overall emotionality and sensitivity of women, which is associated with higher levels of anxiety compared to men^49,50^. Our results are mainly consistent with the results obtained by Martinez-Moreno et al.^51^. It is partially different from the results obtained in the study conducted on children by Grossbard et al.^25^, who found higher scores on the worry subscale but lower scores on the concentration disruption subscale in girls than boys. There were no differences between boys and girls in overall anxiety levels^25^. In our study no differences were also found in the level of anxiety and its components in high-performance and recreational athletes. On the other hand, one might expect that high-performance athletes constitute a group selected in terms of various psychological characteristics, including correlates of emotional resilience^35^. Such a rationale argues for observing a lower level of anxiety in high-performance athletes. In turn, the questions of the questionnaire concern the level of anxiety before or during competition, and high-performance athletes are generally characterized by a greater focus on sporting results, are more subject to personal and social expectations and are subject to social evaluation^52^. These factors may further enhance the experience of anxiety. Hence, it is possible that these effects are somewhat opposite, which is ultimately associated with similar levels of anxiety in the compared groups.

During the proceedings of the validation of the Polish version of SAS-2 scale there were also demonstrated small positive associations of the anxiety (total score, somatic anxiety and concentration disruption) with ego orientation as determined by the TEOSQ questionnaire. Our results were partly similar to the results obtained by the authors of the original scale^9^, although their correlation values were higher, but also not very high. In addition, the relationships obtained by Smith et al.^9^ concerned ego orientation measured by the POSQ questionnaire, which was not observed in our study. Similar relationships—when controlling for social desirability—were also shown by Grossbard et al.^53^ in the study of young athletes. It is possible, as already mentioned in the introduction, that people who are more anxious, anxiously acting—with greater emotional arousal and at the same time with less behavioral control—concentrate more often on confronting the opponent and it is more difficult for them to concentrate, for example, on completing the task. On the other hand, it may be possible that individuals from the sports environment characterized by an ego-enhancing climate simultaneously become more emotionally aroused due to their confrontation orientation and treat the situation as more difficult and threatening^9^. In a more detailed analysis, we could show that the associations of anxiety with ego orientation occurred mainly among recreational athletes, particularly among women (for ego orientation as determined by both the TEOSQ and the POSQ questionnaires). Thus, it is possible that the context of this relation is important here, i.e. the association concerns mainly an environment with lower social expectations of sporting performance (recreational athletes) and among women, who generally present less competitive orientation^54^. In a recreational sport area, there may be greater freedom in the interpretation of the sporting situation—it may be treated more or less confrontationally^55^. Moreover, one’s own expectations of the results may also vary here. Therefore, it is possible that among women participating in recreational sports, there is a large variation in the perception and interpretation of the situation, where women with high expectations of the sporting outcome act more confrontationally (ego orientation) and at the same time experience greater anxiety. Furthermore, no relationship was found between anxiety and task orientation, as noted in previous studies with the SAS-2 scale^9,53^. The results obtained in these aspects are tough to interpret unambiguously and will require further research.

The study is not without limitations, though. Primarily, in further studies on the Polish version of the SAS-2 scale, it is worth extending the research on relations between the SAS-2 and other scales, e.g., another scale measuring anxiety in sport and other variables important from the perspective of functioning in sport, which will additionally broaden the research on the validity of the scale. It is also worth testing the criterion validity of the scale by correlating its results with other variables e.g., various effectiveness indicators in sport. An analysis of differences in anxiety among individual and team athletes would also strengthen the validity of the scale. Although a distinction has been made between high performance and recreational athletes, it would be interesting to conduct validation on high performance athletes with different sport levels ranging from regional to international. Furthermore, the validation has been carried out on relatively young people, so it would be interesting to extend it to different age groups.

The Polish version of the SAS-2 scale has adequate psychometric properties indicating its high validity and reliability, i.e. satisfactory fit of the postulated models to empirical data was demonstrated in confirmatory factor analysis, invariance of the measurement with respect to gender and type of participation in sport was also noted, additionally high reliability indices as assessed by the test–retest method as well as high internal consistency indices were obtained. Women were characterized by lower levels of anxiety than men. Moreover, a negative correlation between anxiety and ego orientation was found, which was particularly evident among women involved in recreational sports activities. These latter issues still require further research. However, the validation results obtained by us support the possibility of using the SAS-2 scale as a valid and reliable tool in scientific research as well as in sports practice. Due to the proceedings of validation conducted by us, the Polish version of SAS-2 scale can be used not only for high-performance, but also for recreational athletes.

## Data Availability

The data generated and/or analyzed during the current study are available from the corresponding author on reasonable request.
